# Kin-Driver: a database of driver mutations in protein kinases

**DOI:** 10.1093/database/bau104

**Published:** 2014-11-19

**Authors:** Franco L. Simonetti, Cristian Tornador, Nuria Nabau-Moretó, Miguel A. Molina-Vila, Cristina Marino-Buslje

**Affiliations:** ^1^Fundación Instituto Leloir, Av. Patricias Argentinas 435. C1405BWE, Buenos Aires, Argentina, ^2^Pompeu Fabra University (UPF), Dept. de Tecnologies de la Informació i les Comunicacions. Tanger 122-140 08018, Barcelona, Spain, ^3^Computational Genomics Laboratory, Genetics Department, Institut de Biologia Universitat de Barcelona (IBUB), Facultat de Biologia, Av Diagonal 645 and ^4^Breakthrough Cancer Research Unit, Dexeus University Hospital, Sabino Arana 5-19, Barcelona, Spain.

## Abstract

Somatic mutations in protein kinases (PKs) are frequent driver events in many human tumors, while germ-line mutations are associated with hereditary diseases. Here we present Kin-driver, the first database that compiles driver mutations in PKs with experimental evidence demonstrating their functional role. Kin-driver is a manual expert-curated database that pays special attention to activating mutations (AMs) and can serve as a validation set to develop new generation tools focused on the prediction of gain-of-function driver mutations. It also offers an easy and intuitive environment to facilitate the visualization and analysis of mutations in PKs. Because all mutations are mapped onto a multiple sequence alignment, analogue positions between kinases can be identified and tentative new mutations can be proposed for studying by transferring annotation. Finally, our database can also be of use to clinical and translational laboratories, helping them to identify uncommon AMs that can correlate with response to new antitumor drugs. The website was developed using PHP and JavaScript, which are supported by all major browsers; the database was built using MySQL server. Kin-driver is available at: http://kin-driver.leloir.org.ar/

## Introduction

Cancer arises due to somatic mutations that result in a growth advantage for the tumor cells. These mutations are known as ‘drivers’ and can be divided into two groups: (i) ‘loss-of-function’ mutations, which inactivate tumor suppressor genes (from here on ‘inactivating mutations’) and (ii) ‘activating’ or ‘gain-of-function’ mutations that transform proto-oncogenes into oncogenes. Somatic mutations in protein kinases (PKs) are frequent driver events in many human tumor types and functionally relevant germ-line mutations are associated with hereditary disorders.

Clinical laboratories worldwide are analysing thousands of human tumor samples, looking for activating mutations (AMs) in certain PKs—such as EGFR, HER2 or BRAF—that correlate with good responses to new generations of antitumor drugs that are kinase inhibitors. Mutations either new or not functionally characterized are often found. In addition, whole-genomic sequencing of human malignancies and other diseases is identifying thousands of changes in PKs, but most of them are likely to be passenger mutations or even polymorphisms. Discriminating driver mutations in PKs is a significant challenge that is hampered by the fact that there are no curated sets of true driver and passenger alterations. The extent of this challenge was evidenced when three state-of-the-art methods, namely MutationAssessor (1), TransFITC (2) and FATHMM (3), were fed with well-established, tumor-associated AMs of PKs and failed to predict them as high impact or disease related (4). Therefore, it is uncertain that the current tools, which are generally based on conservation calculations, can be trusted to screen whole-genome sequencing data in search of driver mutations in PKs. New methods need to be developed and unambiguously assessed datasets of driver mutations are required to train and test them.

## Methods

### Mutation recruitment

Recruitment procedure is described by Molina-Vila *et al*. (4). Briefly, in the case of proto-oncogenic kinases, abstracts and titles of PubMed manuscripts were mined with the kinase name, plus words ‘activating’, ‘gain of function’ or ‘constitutive activation’. For tumor suppressor kinases, the words ‘inactivating’ and ‘loss of function’ were used.

Furthermore, all UniProt entries for human kinases were mined for the same keywords to identify new variants. The references were manually checked to confirm its status.

For each annotated mutation, all samples with that mutation were retrieved from COSMIC using the Biomart perl API.

### MSA construction

Human STK and TKs domains were obtained from Pfam families PF00069 and PF07714, respectively. To account for classification problems in Pfam families, some sequences incorrectly classified as TK were moved from this alignment to the corresponding one and realigned with T-coffee (5). For each MSA, a sequence logo was calculated using seq2logo (6).

### Mutation relative frequency calculation

A relative frequency was computationally calculated for all mutations of the 518 PKs of the COSMIC database release 70 (7) as the frequency of mutation in COSMIC for that gene times 1000 over the total number of tumor samples sequenced for that gene.

All mutations with a relative frequency above 2 (0.2%) were then checked in PubMed by introducing the name of the mutation (e.g. P267R) and added to the dataset if they were found to have functional effects. EGFR mutations conferring a response rate to erlotinib higher than 50%, according to the EGFR somatic mutations database (http://www.somaticmutations-egfr.info/), were also added.

## Results

Kin-Driver database offers a comprehensive set of 560 primary AMs in the kinase and justamembrane (JM) domains of 39 PKs and 83 inactivating mutations in 5 kinases compiled by a two-step systematic search for each of the 518 PKs present in the ‘complete kinase’ study of the COSMIC database (7) (release 70). Only primary mutations with experimental evidence demonstrating their activating/inactivating role were included.

Kin-Driver is a MySQL relational database offering structural and sequence data cross-referenced with COSMIC and with our set of curated mutations. It also provides the frequencies of these mutations in actual tumor samples. The CosmicMart service is used to fetch the data, so frequencies for new mutations can easily be added and data are kept up to date with the periodic COSMIC releases.

Our database can be interrogated by protein name, gene name or keyword, amino acid position or specific mutation name (i.e. T790M). Range or specific mutations can also be used to look for driver mutations in other PKs in equivalent positions (see later). Finally, the database can be browsed by PK name, domain, tissue or type of histology, and these last two attributes obtained from the corresponding mutated samples are available in the COSMIC database.

**Figure 1.**
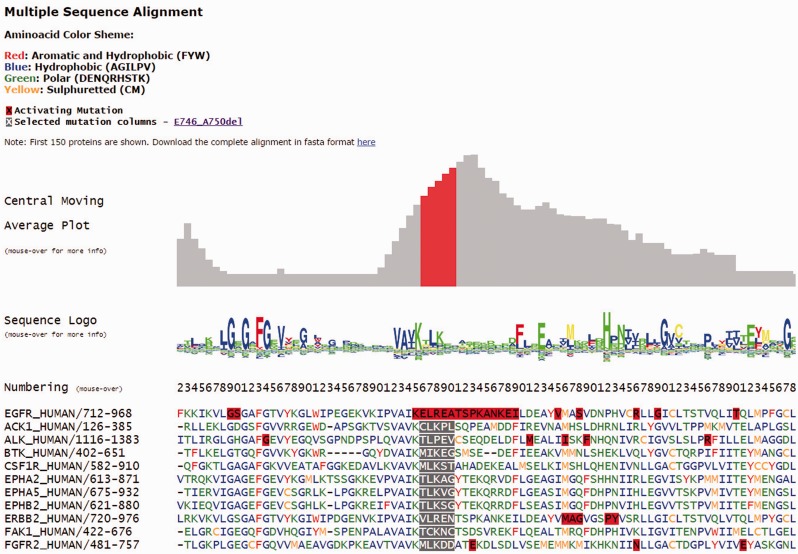
Snapshot of the Kin-Driver EGFR E746-A750del output. MSA logo showing position conservation and MSA highlighting the position of the selected mutation (gray background). Red boxes indicate AMs.

**Figure 2.**
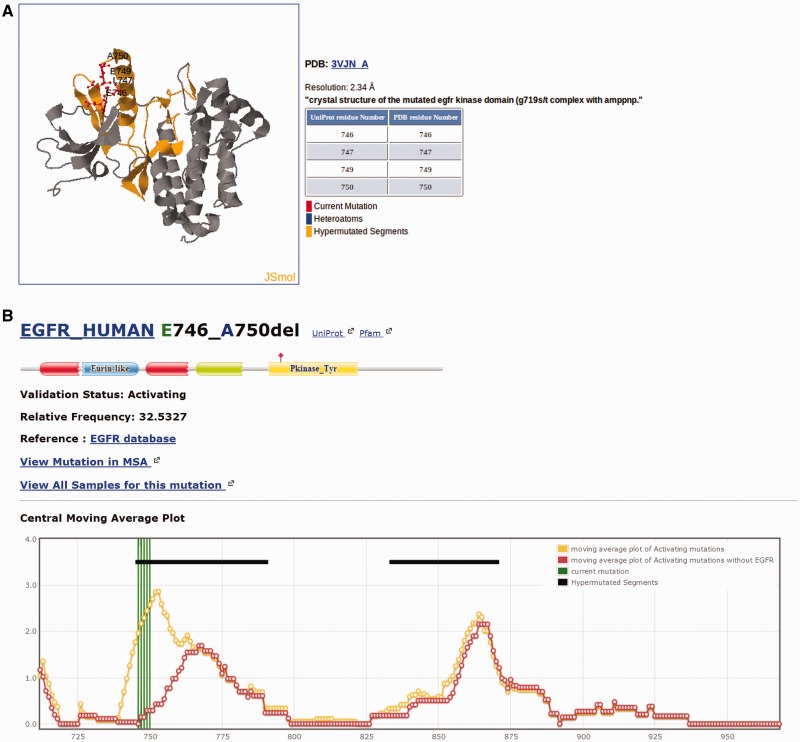
Snapshot of the Kin-Driver EGFR E746-A750del output. (**A**) Mutation EGFR E746-A750del mapped onto the structure of the selected human EGFR (pdb code 1M14). Other structures can be chosen for mapping. (**B**) Location of the mutation in a cmap plot (*n* = 13).

Each individual mutation in Kin-Driver is displayed with its validation status (‘activating’, ‘inactivating’ or ‘unknown’), the mutation type (missense, insertion, deletion, nonsense, frameshift or indel), its absolute and relative frequencies in human tumors and the PubMed reference describing that particular mutation as activating/inactivating. Mutations can also be visualized in a multiple sequence alignment (MSA), placing on top the protein of interest and highlighting the mutation (Figure 1). The PK sequences are classified into one of the two families: tyrosine kinases (TKs, E.C number: 2.7.10.- classification) and serine/threonine kinases (STK, E.C number 2.7.11.-). Each family has its own MSA. The boundaries of the kinase domains are those defined either by Pfam (8) or Uniprot (9). The three-dimensional structure of the protein (when available) marking the mutated position is also shown (Figure 2A). As an example, the output of Kin-Driver for mutation EGFR E746-A750del is presented in Figures 1 and 2.

In case the user challenges Kin-Driver with a particular mutation or position that is not described as activating/inactivating in our dataset, our server retrieves if there is an activating/inactivating mutation in any other human kinase in an equivalent position (based on the MSA alignment). For example, human EGFR in position 724 has no mutation described, but ALK has one AM in the equivalent position G1128A.

Another interesting feature of Kin-Driver is that it incorporates the recent finding that AMs are not randomly distributed within the kinase domain, but cluster in relatively short ‘hyper-mutated’ segments (HSs) (4). In the case of TKs, our server shows the location of the mutation or position interrogated in a central moving average plot (cmap), allowing users to inspect whether it is located within a HS (Figure 2B). The cmap is calculated by adding columnwise all the relative frequencies of all mutations mapped in the MSA obtaining a value per column of the alignment. Then, for each position of the MSA, an average of these values is calculated using a windows size *n* = 13 (Figure 1). Newly discovered mutations, or those of yet unknown effects, that are located within one of the HSs can be suspected to be activating. Therefore, they might be worth further analysis or, if they affect a druggable kinase, might predict a good response to the corresponding antitumor drug (10, 11).

Finally, the possibility to browse Kin-Driver by tissue and histology is also of interest. First, it allows making instant comparisons. For example, it reveals that hematopoietic and lymphoid tumors show the highest frequency of AMs (33.25%), followed by lung (11.96%). For lung tumors, the more frequently mutated kinase is EGFR, with 61 of the 95 described AMs. Second, this kind of browsing can be useful to clinical laboratories; when confronted with a tumor sample, they can use Kin-Driver to find the genes affected by actionable mutations in this particular type of neoplasia, and they can subsequently direct their efforts to the analysis of those genes.

Although mutational data on PKs are currently cataloged in several databases (7, 12–14), they do not discriminate passengers from drivers. Kin-Driver is unique in offering a curated functional annotation, supported by experimental data. Also, the possibility of transferring the mutation to equivalent positions in homologous proteins through an MSA and the mapping into the desired protein structure with the Jsmol app (15) are unique features of our database. In contrast, COSMIC (7) is the most comprehensive compilation of somatic mutations, but no functional status is given, no equivalences between PKs can be inferred, nor the position in the structure is shown. ProKinO (12) provides a kinase ontology and integrates basic mutational data (as recruited from COSMIC) with other types of data, but they do not offer functional or structural information. MoKCa and Canpredict (13, 14) offer a prediction of ‘cancer-associated’ mutations, but it is not based on experimental evidence and the pictures of the mutations in the protein structures are static. Finally, KinMutBase (16) has no information on activation status and is outdated.

## Conclusions

To the best of our knowledge, Kin-Driver is the first comprehensive curated dataset of driver mutations in human cancer. It compiles data otherwise disseminated in several databases that offer poor functional information and in hundreds of articles describing the effects of one or a few mutations each. Our database can be used as a gold standard to develop and validate new bioinformatics methods to predict driver mutations. Second, it sets an easy and intuitive environment for the visualization and analysis of mutations in PKs. And third, it can help researchers and clinicians to recognize relevant mutations in human malignancies.

*Conflict of interest*. None declared.
